# Flummoxed by the flaps – case report

**DOI:** 10.1186/s12877-026-07281-5

**Published:** 2026-03-05

**Authors:** Bhavika Pahwa, Praveen Kumar Tasir, Rohan Savio Sequeira, Milind Khade

**Affiliations:** 1 Grant Government Medical College and Sir JJ Group of Hospitals, Mumbai, Maharashtra India; 2 Department and Institution of Study - Geriatric Department, Grant Medical College and Sir JJ Group of Hospitals, Mumbai, India; 3 Geriatric Department, Grant Government Medical College, Mumbai, Maharashtra India

**Keywords:** Asterixis, Case report, Diabetes mellitus, Hypomagnesemia, Older adult

## Abstract

**Background:**

Asterixis, or “flapping tremor,” is typically described in the setting of metabolic encephalopathies such as hepatic or uremic encephalopathy. However, electrolyte disturbances can also precipitate asterixis, though this is less commonly reported. Hypomagnesemia is an underrecognized cause, particularly in older adults with type 2 diabetes mellitus, where magnesium depletion is multifactorial. Reporting such cases is important to highlight the diagnostic challenge and the potential for fully reversible neurological manifestations.

**Case presentation:**

We describe a 72-year-old woman with long-standing type 2 diabetes mellitus, hypertension, and stage 3 chronic kidney disease, who presented with tingling in all four limbs for 8 months and muscle cramps for 1 month. On examination, she exhibited bilateral asterixis, with otherwise normal neurological findings. Brain imaging showed chronic ischemic changes without basal ganglia involvement, and electrocardiography revealed prolonged QTc. Laboratory studies identified hypomagnesemia (serum magnesium 1.1 mg/dL), while serum calcium and potassium levels were normal. No gastrointestinal losses, drug use, or alcohol history were evident, suggesting diabetes-related magnesium depletion as the underlying cause. After magnesium supplementation, her asterixis resolved completely.

**Conclusions:**

This case emphasizes hypomagnesemia as a rare but reversible cause of asterixis. In older adults with diabetes, magnesium depletion is multifactorial and underdiagnosed. Early recognition and correction are crucial to prevent complications.

## Background

Flapping tremors, or asterixis, are commonly associated with hepatic encephalopathy, renal insufficiency, and respiratory failure [1]. However, they can result from multiple metabolic and structural causes. Hypomagnesemia, although frequently overlooked, is a clinically important electrolyte disturbance that can present with diverse neurological, cardiovascular, endocrinal and muscular manifestations. Despite this, presentations such as asterixis attributable solely to hypomagnesemia are very uncommon. A limited number of individual case reports have implicated low magnesium as a primary cause of asterixis in the absence of concurrent hypokalaemia or hypocalcaemia. Describing such cases is valuable in reinforcing magnesium deficiency as a potentially reversible contributor to disabling neurological symptoms.

This case underscores the need to evaluate asterixis beyond its usual aetiologies. It demonstrates isolated hypomagnesemia as a rare yet reversible cause of asterixis in an older adult with type 2 diabetes, highlighting the critical role of early recognition and correction in avoiding unnecessary investigations and complications.

## Case report

A 72-year-old woman with type 2 diabetes mellitus, hypertension and diabetic nephropathy presented with complaints of - tingling sensation in all four limbs for 8 months and muscle cramps and spasms for 1 month to neurology. On evaluation, bilateral flapping tremors were observed for last 2 days and she was referred to geriatric outpatient department for suspicion of uremic encephalopathy.

On further evaluation patient reported that her urine output was 1–1.5 L per day with no history of any reduction or change in it. There were no symptoms of uraemia.

On examination she was conscious, co-operative and oriented to time, place and person. Her vitals and general physical examination were normal. Bilateral flapping tremors were present. No other abnormal movements such as tremor, myoclonus, or abnormal eye movements like nystagmus or opsoclonus were observed. On neurological examination – higher mental function and cranial nerve examination were normal; bulk, power, tone, deep tendon reflexes and co-ordination were normal, bilateral plantar reflex were flexor. Sensory examination was normal. No signs and symptoms of meningitis were present. Table 1 shows her investigation results.

**Table 1:** Serum and Urine Analysis of the patient

**Parameter**	**Result**	**Reference range**	**Reference Unit**
Haemoglobin	11.3	12–15	gm/dL
White-cell count	9,630	4000–10,000	/mm³ (per cubic mm)
Platelets	3.7	1.5–4.1	lakh/µL
Serum total bilirubin	0.3	0.2–0.8	mg/dL
Serum albumin	3.6	3.5–5	gm/dL
ALT (Alanine aminotransferase)	19	< 50	IU/L
AST (Aspartate aminotransferase)	25	< 50	IU/L
Blood urea	65	15–40	mg/dL
Serum creatinine	1.4	0.5–1.2	mg/dL
eGFR (Estimated Glomerular FiltrationRate)	40	90–120	mL/min/1.73 m²
Serum sodium	142.6	135–145	mmol/L
Potassium	4.57	3.5–5.0	mmol/L
Chloride	103.9	95–105	mmol/L
Fasting blood sugar	118	70–100	mg/dL
Post-prandial blood sugar	225	< 140	mg/dL
Glycosylated haemoglobin (HbA1c)	6.76	< 5.7	%
Urine microscopy	Proteinuria 2+ No glycosuria, no bacteria	Negative – 4+	
TSH	0.682	0.35–5.5	µIU/mL
T3	0.89	0.7–2.04	ng/mL
T4	7.36	5.1–14.1	µg/dL
Serum Triglycerides	116	30–150	mg/dL
Total Cholesterol	199	130–230	mg/dL
HDL Cholesterol	59	40–60	mg/dL
LDL Cholesterol	23	12–35	mg/dL

In this patient with pre-existing renal disease, we would likely attribute the cause of asterixis to the renal failure. But asterixis are seen in advanced untreated kidney failure, while this patient is in stage 3 CKD without any signs of worsening. So, she was evaluated for various other causes of asterixis (Table 2) [1, 2]. 

**Table 2:** Causes of bilateral asterixis

Metabolic: Liver failure, azotaemia, respiratory failure
Electrolyte abnormalities: Hypomagnesemia, hypokalaemia
Bilateral structural brain lesions
Medications Causing Asterixis Antibiotics: Ceftazidime Sedatives: Benzodiazepines, barbiturates Antipsychotics: Lithium Anticonvulsants: Phenytoin (phenytoin flap), carbamazepine, valproic acid, gabapentin Other drugs: Metoclopramide, anticholinergics, opiates, levodopa

Her medication history suggested she was taking tablet Metformin 500 mg twice daily, tablet Glimepiride 2 mg once daily, tablet Metoprolol 25 mg once daily, tablet Telmisartan 40 mg once daily, tablet Aspirin 75 mg once daily, tablet Atorvastatin 20 mg at nighttime and tablet Sodium bicarbonate 500 mg twice daily. There was no history of use of any anticonvulsants, sedatives or antipsychotic drugs.

A non-contrast brain computed tomography revealed chronic small vessel ischemic changes in bilateral frontoparietal lobes involving bilateral centrum semiovale, corona radiata and periventricular white matter. No involvement of basal ganglia, internal capsule or thalamus was detected.

Her electrocardiogram showed a prolonged QTc interval of 516 milliseconds, prompting evaluation of serum calcium and magnesium levels. The results revealed a serum calcium of 10 mg/dL (corrected calcium was 10.32 mg/dL) and a serum magnesium of 1.1 mg/dL.

Based on the evaluation it became evident that the hypomagnesemia was causing asterixis in this patient. Following which she was admitted and given bolus dose of intravenous magnesium sulphate of 2 g under telemetry. She underwent further evaluation for the cause of hypomagnesemia while simultaneously receiving treatment for it. Glycaemic control was also done.

In this patient there was no history of any recent use of PPI or loose stools or any abdominal complaints. Her 24-hour urinary magnesium was 157 mg, suggesting renal loss of magnesium ion as cause for hypomagnesemia. A 24-hour urinary magnesium excretion of > 24 mg is consistent with renal causes of hypomagnesemia [3]. 

An ABG was done which was suggestive of a pH of 7.43, bicarbonate of 26.2 mmol/L, pCO2 of 40.3 mmHg. Her 24-hour urinary calcium was 100 mg/24 hours (range 100–300). Table 3 shows her urinary electrolytes.

**Table 3:** Spot Urine Creatinine and Electrolytes Panel

**Urinary electrolytes**	**Result**	**Reference Range**	**Unit of Range**
Urinary creatinine (spot)	50.14	24–275	mg/dL
Urinary sodium (spot)	115	15–237	meq/L
Urinary potassium (spot)	23	22–164	meq/L
Urinary chloride (spot)	130	24–255	meq/L
Urinary calcium (spot)	5.7	0.5–37.5	mg/dL

Normal serum and urinary electrolyte levels, aside from magnesium, indicated an isolated magnesium deficiency in this patient. Ruling out causes of hypomagnesemia with salt wasting. There was no history of use of diuretics or aminoglycosides or any chemotherapeutic and nephrotoxic drugs. The absence of failure to thrive during infancy, seizure episodes in childhood, and any prior history of magnesium supplementation made inherited causes less likely. There was also no history of alcohol or substance use. Consequently, her hypomagnesemia could be attributed to diabetes mellitus–related renal magnesium wasting.

Patients with diabetes mellitus often develop hypomagnesemia, which has been attributed to the role of insulin in regulating TRPM6 activity and expression. Other mechanisms include hyperfiltration, increased urinary flow, and decreased intestinal absorption due to autonomic neuropathy [4]. 

She was given intravenous infusion of magnesium. Followed by maximum tolerated oral magnesium. Her asterixis improved at serum magnesium of 1.3 mg/dl. Even after 2 months of oral supplementation her serum magnesium remained low at 1.3 mg/dl so she was also started on Dapagliflozin 10 mg orally once a day. Following which her serum magnesium levels stabilized at 1.7 mg/dl. On follow up after two months of oral magnesium tablet, dapagliflozin and diet rich in magnesium her magnesium level was 2.2 mg/dl.

## Discussion

Flapping tremors or asterixis has many structural and systemic causes but most physicians are familiar with them in context to hepatic encephalopathy, renal insufficiency and respiratory failure [2]. The management of asterixis is dependent on the underlying pathology therefore when a patient presents with asterixis without common metabolic causes like in this case, a thorough search for varied causes at varied locations should be done [1]. 

The exact mechanism of generation of asterixis remains unknown in hypomagnesemia. It has been suggested that fluid shifts result in swelling of Alzheimer type II astrocytes and associated metabolic abnormalities. These alterations disrupt the blood–brain barrier, leading to upregulation of peripheral benzodiazepine receptors and increased neurosteroid production. These cause abnormal function of diencephalic motor centres that regulate the agonist and antagonist tones. Nonetheless, how these alterations specifically give rise to asterixis and why this neural circuitry is especially susceptible remain uncertain [2]. 

Older adults are more susceptible to electrolyte abnormalities. Bedside electrocardiography (ECG) serves as an important aid in forming diagnoses in such patients. Although isolated hypomagnesemia does not have any classical ECG presentation it is associated with longer P wave duration, QTc, Tpec interval (corrected T peak-to-end interval) and Tpe/QT ratio, indicating increased dispersion of ventricular repolarization [5, 6]. Therefore though not very specific but prolonged QTc prompted screening for additional electrolyte disturbance in this case.

Hypomagnesemia may manifest with neurological symptoms such as paraesthesia, seizures, and altered mental status; cardiovascular complications including dysrhythmias and vasospasms; endocrine disturbances like hypokalaemia and hypocalcaemia; and muscular issues such as bronchospasm and muscle weakness [6]. In symptomatic patients, plasma magnesium levels are often significantly reduced. However, serum concentrations may not accurately indicate the extent of magnesium depletion, as the majority of magnesium is located intracellularly. Isolated hypomagnesemia is unusual. Magnesium deficiency is usually part of a complex electrolyte imbalance, often accompanied by hypokalaemia, because of increased potassium secretion in the distal nephron, and hypocalcaemia because of reduced responsiveness to parathyroid hormone (PTH) as well as reduced PTH levels. In this patient the plasma potassium concentrations may not be low due to hyperkaliaemic effect of telmisartan. Given the importance of accurate diagnosis and holistic management for recovery, the patient was evaluated for additional electrolyte disturbances and underlying causes of hypomagnesemia [7, 8]. 

Hypomagnesemia is induced by two major mechanisms: gastrointestinal or renal losses.

Gastrointestinal losses include marked dietary deprivation, diarrhoea, malabsorption, pancreatitis, the chronic use of proton pump inhibitors, and primary familial hypomagnesemia. Urinary magnesium losses can be because of medications including thiazide diuretics, aminoglycoside, foscarnet, chemotherapeutic and immunosuppressive agents; inherited disorders; hypomagnesemia with salt wasting (Gitelman syndrome, Bartter syndrome and hypomagnesemia with hypocalcaemia syndromes); alcohol use disorder and diabetes mellitus [4, 8] In our case diabetes mellitus–related renal magnesium wasting was found as the likely cause for her hypomagnesemia.

The prevalence of hypomagnesemia is approximately 2% in the healthy population. In Type 2 diabetes, cohort studies report a prevalence of hypomagnesemia between 9.1% and 47.7%. Henceforth, hypomagnesemia occurs about ten times more frequently in diabetics than in the general population and is linked to insulin resistance, hyperglycaemia, and accelerated disease progression. In contrast, hypomagnesemia is uncommon in type 1 diabetes, indicating that insulin resistance plays a central role in disrupted magnesium homeostasis. Age, poor glycaemic control, low estimated glomerular filtration rate (eGFR) and insulin resistance are considered to be significant determinants of hypomagnesemia in T2D [9]. In our patient the Triglyceride-Glucose Index (TyG) was 4.76. TyG index cutoff of 4.68 has a sensitivity of 96.5% and a specificity of 85% in estimating insulin resistance [10]. 

There is a limited data and no organized consensus guidelines to guide management in patients with hypomagnesemia. Since there were no life-threatening symptoms such as seizures or ventricular arrhythmias, the intravenous formulation was administered at a rate of 1 g per hour to enhance magnesium retention following the bolus dose. There is a lack of robust empirical evidence on the oral bioavailability of various magnesium salts. However, it is generally assumed that organic salts have higher bioavailability than their inorganic counterparts. A reasonable target dose is to administer 300 mg of elemental magnesium in divided doses and titrate as tolerated [4]. So according to patients’ tolerance she was given 400 mg Magnesium oxide tablet (241.3 mg elemental magnesium) twice daily. The patient was advised to increase her consumption of magnesium-rich foods, such as seeds, nuts, and legumes. Still patients’ magnesium levels remained low. Randomized controlled trials of sodium-glucose cotransporter 2 (SGLT2) inhibitors have shown that patients receiving these treatments had higher plasma magnesium levels compared to those given a placebo. Multiple case studies also support this finding [4, 11]. A few potential mechanisms by which SGLT2 inhibitors may influence Mg²⁺ homeostasis include enhanced sodium delivery to the thick ascending limb, which increases the transepithelial membrane potential through upregulated activity of the Na⁺-K⁺-2Cl⁻ cotransporter (NKCC2), thereby facilitating passive paracellular Mg²⁺ reabsorption. Additionally, elevated levels of epidermal growth factor (EGF), glucagon, uromodulin, and tubular flow along with activation of the Na⁺-Cl⁻ cotransporter and reductions in insulin resistance and reactive oxygen species can directly promote TRPM6 (transient receptor potential cation channel subfamily M member 6) mediated transepithelial Mg²⁺ transport in distal convoluted tubule [12]. So she was started on tablet Dapagliflozin 10 mg once a day. Following which improvement was seen. The patient continued to maintain magnesium levels on oral magnesium tablet, dapagliflozin and diet rich in magnesium.

This case highlights the importance of evaluating for underlying causes of asterixis, as well as the predisposition of individuals with diabetes mellitus to renal magnesium wasting.

## Data Availability

Yes, all data generated or analysed during this study are included in this published article.
